# HPLC-based activity profiling for pharmacologically and toxicologically relevant natural products – principles and recent examples

**DOI:** 10.1080/13880209.2019.1606261

**Published:** 2019-05-06

**Authors:** Matthias Hamburger

**Affiliations:** Pharmaceutical Biology, Pharmacenter, University of Basel, Basel, Switzerland

**Keywords:** GABAA receptor, hERG, *Piper nigrum*, *Evodia rutaecarpa*, piperine, dehydroevodiamine, hortiamine

## Abstract

**Context:** Discovery of pharmacologically active natural products as starting points for drug development remains important and, for reasons of consumer safety, the identification of toxicologically relevant compounds in herbal drugs.

**Objective:** To explain, with the aid of relevant examples from our own research, how these goals can be achieved.

**Methods:** An in-house technology platform comprising pre-formatted extract libraries in 96-well format, miniaturized tracking of activity in extracts via HPLC-activity profiling, structure elucidation with microprobe NMR, and *in vitro* and *in vivo* pharmacological methods were used.

**Results:** Piperine was identified as a new scaffold for allosteric GABA_A_ receptor modulators with *in vivo* activity that interacts at a benzodiazepine-independent binding site. Selectivity and potency were improved by iterative optimization towards synthetic piperine analogues. Dehydroevodiamine and hortiamine from the traditional Chinese herbal drug Evodiae fructus were identified as potent hERG channel blockers *in vitro*. The compounds induced torsades de pointes arrhythmia in animal models.

**Conclusions:** The allosteric binding site for piperine analogues remains to be characterized and cardiac risks of herbal drugs need to be further evaluated to ensure consumer safety.

## Introduction

The relevance of natural products for drug discovery and development is undisputed. In the nineteenth and early twentieth century, drug substances were mainly natural products, but during the twentieth century derivatives of natural products and natural product-inspired fully synthetic drug substances became increasingly important. But even in recent years approximately 35% of the new chemical entities approved as drugs were directly derived from natural products (Newman and Cragg 2012). Despite this impressive track record, a steady decline of interest for natural products has occurred within the pharmaceutical industry. Several factors have led to this apparently paradoxical situation, for example, the advent of new technologies such as combinatorial chemistry which appeared at one time more promising and ‘easier’ for generating screening libraries. Also, with the triumph of high throughput screening (HTS) in the 1990s, natural product research struggled to find its place in the rapidly evolving drug discovery landscape. It became increasingly clear that the classical approach of preparative bioactivity-guided fractionation was incompatible with the fast turnaround and tight deadlines of modern screening programs (Potterat and Hamburger 2013).

Virtually all major pharmaceutical companies have meanwhile discontinued their in-house natural product discovery programs. However, tremendous technological advancements in chromatography and spectroscopy enable entirely new ways as to how natural products can be investigated. These novel opportunities in natural product research have been seized by a number of startup companies, and by some academic research groups. Strategies combining, in a more or less seamless manner, libraries of extracts or fractions with HPLC microfractionation, spectroscopic and bioactivity data allow for early prioritization of hits, and identification of bioactive constituents without the need for preparative amounts of material (Camp et al. 2012; Potterat and Hamburger 2013). Bioinformatics tools enable the annotation of extract libraries, thereby enhancing the value of such resources and accelerating the discovery process (Allard et al. 2017; Da Silva et al. 2018).

We established 15 years ago in our laboratory a technology platform for library-based lead discovery, drawing on previous experience in an industrial natural products HTS operation. Key elements of the platform are (i) an extract library in 96-well format, (ii) a customized database for sample management, (iii) automated liquid handling, (iv) HPLC-based tracking of bioactivity via microfractionation for off-line bioassays and (v) on-line (DAD, ESI- and APCI-MS, including HR-MS) and off-line (microprobe NMR) spectroscopy for structure elucidation (Potterat and Hamburger 2014).

The extract library currently comprises over 4000 extracts, mostly of plant origin. The dissolution of extracts in DMSO, at a concentration of 10 mg/mL, and the subsequent transfer to the storage plates are carried out by a robotic system. The extract library is formatted in racked 2D-barcoded microtubes in 96-well format, and several copies of the library have been prepared and are stored at –80 °C. One copy serves for ongoing screening and profiling activities, and the other copies for long-term backup. When samples are to be delivered to a screening project, the library, or specific portions of it, are replicated into 96-well daughter plates.

## HPLC-based activity profiling

**Figure 1.**
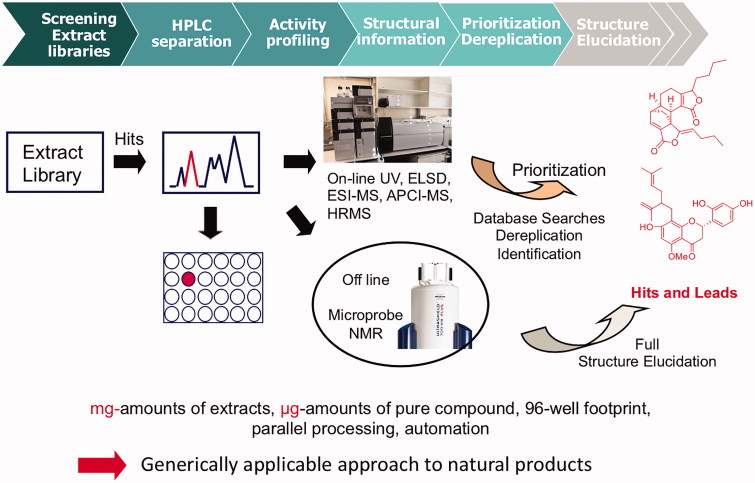
Platform for natural product-based lead discovery.

The efficient tracking of the active compound(s) in an extract remains the single most challenging step in the discovery process. The tedious and slow preparative bioactivity-guided isolation is no more compatible with the requirements of modern drug discovery. Also, loss of activity during purification, and repeated isolation of the same compounds occur frequently. More efficient alternatives have been developed to track bioactivity in complex samples (Potterat and Hamburger 2013). Of these, HPLC-based activity profiling has emerged as a highly versatile strategy to miniaturize and accelerate identification of active compounds and is routinely used in our lab (Potterat and Hamburger 2014) (Figure 1). Bioactive extracts are separated by analytical (or semi-preparative) HPLC, with UV and MS data recorded on-line. In parallel, the column effluent is fractionated into deep-well plates via a T-split. After drying of plates, they are sealed and shipped for screening. Prior to the bioassay, microfractions are redissolved in a small volume of a suitable solvent (typically DMSO) and diluted. An overlay of the HPLC chromatogram and the activity profile pinpoints to active peaks, while on-line spectroscopic information in combination with database searches allows to either dereplicate known compounds and/or highlight potentially new molecules. We usually perform the microfractionation step directly with the library sample, since mg amounts of extracts are sufficient for the purpose. Subsequent preparative purification is done after extraction of plant material at larger scale, and with a peak-guided strategy using PDA or MS (with a T-split).

For a successful implementation of HPLC-based activity profiling, some practical aspects are critical: the choice of the HPLC column to be used depends on the degree of miniaturization and sensitivity of the bioassay. For most cellular and biochemical assays, separation on an analytical HPLC column (3 mm i.d.) is sufficient, and we typically inject 300 µg of extract (corresponding to 30 µL of DMSO solution). For few pharmacological assays, the separation has to be performed on a semi-preparative scale. This is the case, for example, with assays utilizing *Xenopus* oocytes (e.g., GABA_A_ and hERG assays that will be discussed later) for which aliquots of 5 mg of extract are separated on a semipreparative column (i.d. 10 mm) (Kim et al. 2008; Potterat and Hamburger 2014).

Prior to using a given bioassay, its suitability for the purpose needs to be checked. Compounds in the extract may interfere with the assay readout, and low sensitivity of the assay or a poor signal-to-noise ratio may be limiting factors. Validation of an HPLC activity profiling protocol involves runs with pure active substances, microfractionation of inactive extracts spiked with a known active compound, and profiling of active extracts containing known active compounds (Kim et al. 2008; Moradi-Afrapoli et al. 2017). Plant extracts often contain tannins which are likely interference in many bioassays, and not only in biochemicals screens with purified proteins. In case of positive assay results suspected to be due to tannins, SPE of the extract over a polyamide cartridge and re-profiling is performed (Grabher et al. 2012). HPLC activity profiles not only locate the activity in the extract, but also provide additional important information for prioritization of samples for a preparative follow-up. Preliminary structure–activity data are a further type of information which can be obtained by extending the on-line spectroscopic analysis beyond the active compounds towards inactive, but structurally related molecules (Potterat and Hamburger 2014).

## Discovery of novel leads – GABA_A_ receptor modulators

Over the years we have validated and used, mostly in collaborative projects, HPLC profiling protocols for a wide range of biological targets and assay formats (Potterat and Hamburger 2013, 2014). One of our discovery projects was aiming at novel allosteric modulators of γ-aminobutyric acid type A (GABA_A_) receptors which are the major inhibitory neurotransmitter receptors in the mammalian brain. GABA_A_ receptors are heteropentamers forming a central chloride-conducting pore. A total of 19 subunits (α1–6, β1–3, γ1–3, δ, ε, θ, π and ρ1–3) are known which can assemble into receptor subtypes that differ in their distribution in the CNS, and in their sensitivity to GABA and various drugs (Sieghart and Sperk 2002; Simon et al. 2004; Barrera and Edwardson 2008). The most abundantly occurring receptor subtype is formed of two α1, two β2 and one γ2 subunits (α_1_β_2_γ_2_ receptor) (Olsen and Sieghart 2008). Drugs, such as benzodiazepines, barbiturates, neurosteroids and anaesthetics enhance chloride currents through GABA_A_ receptors and play an important role in the treatment of general anxiety, panic disorders, sleep disturbances, epilepsy or for anaesthesia (Whiting 2006; Riss et al. 2008). However, these drugs show poor selectivity towards specific receptor subtypes, and drugs such as the widely used benzodiazepines therefore possess a spectrum of side effects including dependence, unwanted sedation and amnesia.

**Figure 2.**
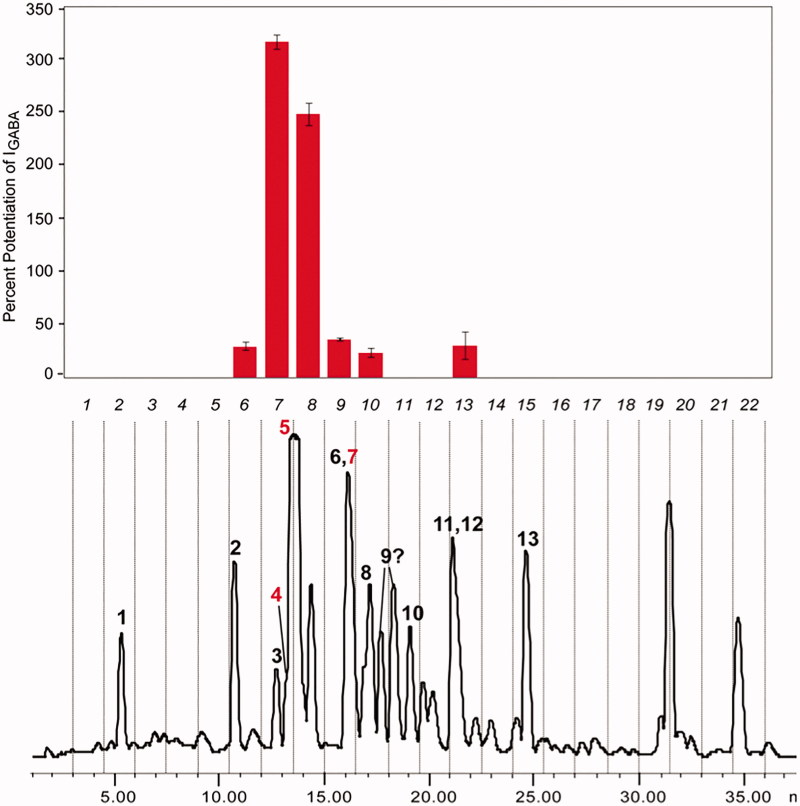
HPLC activity profiling of an EtOAc extract of *Piper nigrum* fruits. The HPLC chromatogram (254 nm) of a semipreparative separation of 5 mg of extract is shown, with the activity (potentiation of I_GABA_) of microfractions of 90s each displayed above. Peak numbering corresponds to identified piperamides, with red numbers highlighting active compounds piperanine (**4**), piperine (**5**) and weakly active piperettine (**7**). Separation was on a Sunfire RP18 column (5 µm, 10 × 150 mm); 30–100% MeCN in 30 min; 4 mL/min.

From a screening of our extract library, we identified piperine as an allosteric GABA_A_ receptor modulator (Zaugg et al. 2010) (Figure 2). This well-known compound was interesting as a lead insofar as it showed *in vivo* activity in rodents in behavioural paradigms, had favourable physicochemical properties, and represented a new scaffold of allosteric GABA_A_ receptor modulators interacting at a benzodiazepine-independent binding site (Zaugg et al. 2010; Khom et al. 2013). On the downside, the compound was known to be pharmacologically promiscuous, with activity at the transient receptor potential vanilloid type 1 (TRPV1) receptor, and interaction with drug transporters and metabolizing enzymes.

**Figure 3.**
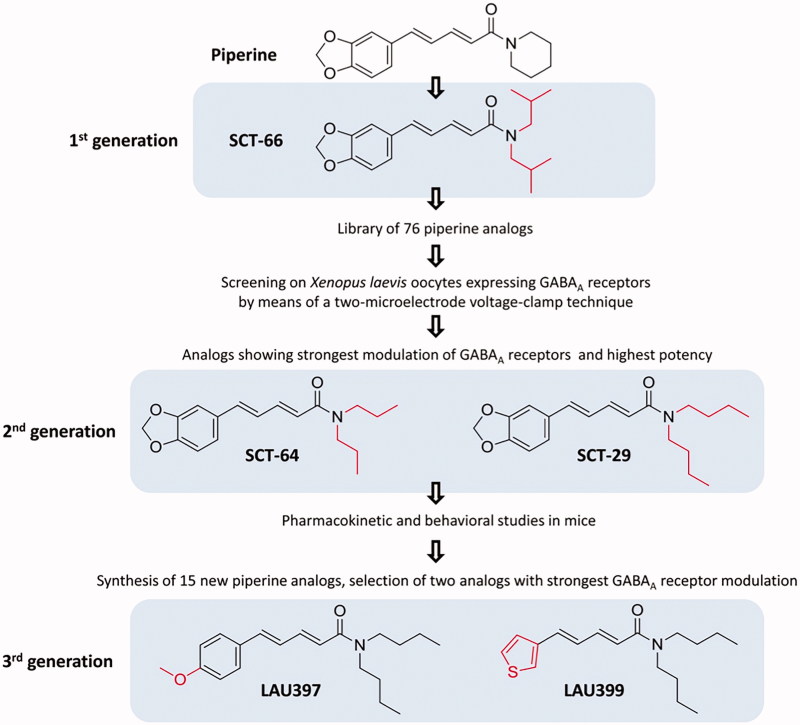
Key steps in the development of piperine to fully synthetic analogues.

From the HPLC-activity profiling, some preliminary structure–activity information regarding GABAergic activity could be derived. The length and rigidity of the spacer between the piperidine and the aromatic ring appeared to be relevant for activity, as well as the nature of the amide moiety (Zaugg et al. 2010). This information was taken into account when a medicinal chemistry project on piperine analogues was established (Figure 3). A first critical finding was that activities at GABA_A_ and TRPV1 receptors could be separated in semisynthetic analogues. Replacement of the piperidine ring by an *N*,*N*-diisobutyl residue led to (2*E*,4*E*)-5(1,3-benzodioxol-5-yl)-*N*,*N*-diisobutyl-2,4-pentadienamide (SCT-66) which showed markedly reduced interaction with TRPV1 receptors. SCT-66 enhanced chloride currents through GABA_A_ receptors more potently and efficiently than piperine, and exhibited a stronger anxiolytic action *in vivo* (Khom et al. 2013). Based on these findings, a library of piperine derivatives was synthesized and investigated with respect to modulation of α_1_β_2_γ_2S_ GABA_A_ receptors expressed in *Xenopus laevis* oocytes. Modifications at the amide functionality and on the diene motif of piperine were emphasized in order to enhance the modulatory potential of analogue structures (Schöffmann et al. 2014).

The activity at GABA_A_ and TRPV1 receptors was evaluated, and SCT-29 and SCT-64 were selected for assessment of *in vivo* activity and for snapshot pharmacokinetic analysis, whereby the *N*,*N*-dibutyl derivative SCT-29 was superior. In a next step, a series of synthetic analogues were prepared in which the 1,3-benzodioxol moiety was replaced, considering the fact that such moieties could possibly be metabolized by CYP450 isoenzymes to *o*-quinones which may alkylate cellular nucleophiles and/or form reactive oxygen species through redox cycling (Bolton et al. 1997). Lau397 and Lau399 showed the highest activity at GABA_A_ receptors (Wimmer et al. 2015). Key compounds from the three cycles of optimization were then submitted to *in vitro* assessment of biopharmaceutical properties, whereby measurement of blood–brain barrier (BBB) permeability (Eigenmann et al. 2016), Caco-2 permeability, plasma protein binding, *in vitro* metabolic stability in microsomal incubations, CYP450 phenotyping and tentative metabolite identification were performed, together with a snapshot pharmacokinetic study in mice (Zabela et al. 2018). While BBB permeability was acceptable for piperine and SCT-64, all compounds underwent rapid phase I metabolization, with involvement of different CYP450 isoenzymes. Ongoing optimization is aiming at improving metabolic stability while maintaining good BBB permeability.

## Characterization of compounds with undesired effects – hERG channel blockers in herbal drugs

An increasing number of non-antiarrhythmic drugs have been found to exhibit side effects associated with a prolongation of the QT interval in the electrocardiogram (Sanguinetti and Tristani-Firouzi 2006). Drug-induced long QT syndrome (LQTS) may lead to ventricular tachyarrhythmia (Torsades de Pointes (TdP) arrhythmia) and sudden cardiac death. Drugs such as terfenadine and cisapride were withdrawn from the market due to this potentially fatal side effect. The most important determinant of acquired LQTS is inhibition of *I*_Kr_ (*I*_hERG_), the rapidly activating component of the delayed rectifier potassium current that is mediated by the hERG (human ether-a-go-go-related gene) channel. Reduction of *I*_hERG_ delays the repolarization phase of the cardiac action potential and, as a consequence, leads to prolongation of the QT interval (Dennis et al. 2007). hERG channel blockage is considered as a major safety liability in preclinical drug development and clinical practice and is, therefore, routinely assessed for drug leads and drug candidates in industrial drug discovery and development (Hancox et al. 2008; Gintant 2011). Considering the importance of hERG as an antitarget, surprisingly few natural products have been tested for hERG channel blocking properties. When we initiated our hERG project, a limited number of natural products only had been shown to diminish hERG channel activity *in vitro* (Zitron et al. 2005; Kim et al. 2008; Jeong et al. 2010; Xing et al. 2010; Harmati et al. 2011; Hu et al. 2012; Liu et al. 2012). The possible clinical relevance, however, had been highlighted by the fact that the consumption of 1 L of freshly squeezed pink grapefruit juice (containing naringenin, a weak hERG channel blocker) led to a mild prolongation of the QTc interval in both young healthy volunteers and patients suffering from cardiomyopathy (Zitron et al. 2005; Piccirillo et al. 2008). Furthermore, the hERG channel inhibitory effect of the anti-arrhythmic drug amiodarone was potentiated *in vitro* by simultaneous administration of naringenin (Lin et al. 2008). The fact that widely occurring natural products could block *I*_hERG_ in humans led us to screen medicinal and dietary plants for their *I*_hERG_ inhibitory potential.

A library of extracts prepared from European and Asian medicinal plants, vegetables and spices was screened, along with a library of structurally diverse alkaloids (Schramm et al. 2014a, 2014b). Some of the alkaloids tested exhibited mild, but non-significant hERG inhibition. As for the medicinal plants and spices, an extract from Coptidis rhizoma (rhizome of *Coptis sinensis*) showed some activity, and hERG inhibition was tracked to the moderately active dihydroberberine (Schramm et al. 2011). The by far most potent inhibition was found with Evodiae fructus. This herbal drug consists of the nearly ripe, dried fruit of *Evodia rutaecarpa* (Rutaceae ), and is among the popular herbal drugs in traditional Chinese medicine (TCM) (Schramm and Hamburger 2014).

Indoloquinazoline and quinolone alkaloids, flavonoids and limonoids have been previously identified in the drug (Sugimoto et al. 1988; Wang YF et al. 2011; Huang et al. 2012; Wang XX et al. 2013). Of these, dehydroevodiamine (DHE, **1**), a major secondary metabolite in *Evodia* fruits, has been widely studied from a pharmacological point of view. Among others, vasorelaxant effects have been reported (Chiou et al. 1996), and electrophysiological studies in isolated guinea pig cardiomyocytes revealed that DHE inhibited several cardiac ion currents (e.g., *I*_Na_, *I*_Ca,L_ and *I*_K_) and prolonged duration of the atrial and ventricular action potential (Yang HY et al. 1988; Lin et al. 1990; Yang MCM et al. 1990).

**Figure 4.**
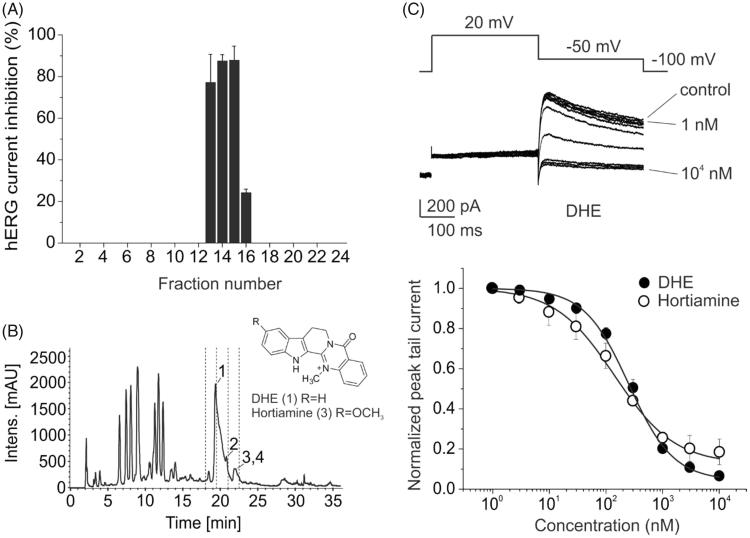
HPLC activity profiling of *Evodia rutaecarpa* MeOH extract. (A, B) HPLC chromatogram (204 nm) of a separation of 5 mg extract and inhibition of hERG tail currents by microfractions. Structures of active compounds dehydroevodiamine (DHE) (**1**) and hortiamine (**3**) are shown. (C) Currents through hERG channels expressed in HEK 293 cells in absence (control) and presence of increasing concentrations of DHE during 0.3 Hz pulse trains are shown above, together with the voltage protocol. Concentration dependent inhibition of potassium current by DHE and hortiamine during repeated pulsing at 0.3 Hz at a holding potential of –100 mV is shown below. Reprinted with permission from Baburin et al. (2018).

By HPLC-activity profiling and subsequent preparative isolation the indoloquinazoline alkaloids DHE and hortiamine were identified as potent hERG inhibitors in *Evodia* extract, with IC_50_ of 253.2 ± 26.3 nM and 144.8 ± 35.1 nM, respectively, in patch clamp experiments (Figure 4). DHE prolonged the action potential duration in dog ventricular cardiomyocytes in a concentration dependent manner and induced early after depolarizations. A proarrhythmic potential of DHE was confirmed in anesthetized rabbits and chronic atrioventricular block (cAVB) dogs (0.33–0.5 mg/kg/5 min) where the drug increased the QT interval and induced TdP arrhythmia. Interestingly, a higher dose did not induce TdP. hERG channel block by DHE and hortiamine was dramatically reduced by mutations of the two know putative binding determinants on the S6 segments (Y652, F656). Docking studies suggested that DHE binds to the central cavity of hERG and forms hydrophobic interactions with Y652, and π–π-stacking interactions with the aromatic rings of F656. It seems that the indole ring of DHE is able to arrange itself between Y652 and F656 in a sandwich type manner, thereby allowing π–π stacking interactions with both amino acids (Baburin et al. 2018).

We determined the DHE content in *Evodia* herbal drug samples from different suppliers, and in *Evodia* extract-containing granules that are sold as food supplements. Taking into consideration the recommended daily doses for herbal drug and granules, a possible daily intake of DHE of up to 40 mg was calculated (Schramm and Hamburger 2014). To further substantiate the relevance of our findings, we determined the amount of DHE that was extracted when preparing herbal drug decoctions according to TCM procedure. We found that approx. 25% of the DHE content was extracted which still would correspond to a significant daily intake of the alkaloid. The hERG channel blocking properties of the herbal decoctions was proportional to their DHE content (unpublished results).

## Conclusions

HPLC-based activity profiling is an efficient means for tracking of active compounds in an extract. Typically, it is performed at the scale of analytical HPLC, and localization of the activity in the extract is the primary focus. Scale-up to semi-preparative or preparative HPLC enables rapid purification of compounds in the active time window for analysis by microprobe NMR. However, the utility of HPLC-based activity profiling is not limited to the mere localization and identification of bioactive peaks, as it can provide a wealth of additional and highly useful information. First, activity profiles are extremely important for prioritization of active extracts, since screening of a large extract library quite often delivers a number of hits exceeding the capacities for a preparative follow-up. Spectroscopic data recorded on-line provide valuable structural information for dereplication of known compounds. Second, preliminary structure–activity information can be obtained which may be useful for further optimization of compounds by medicinal chemists, as highlighted with the case of piperamides. Natural products may not necessarily possess all properties required for a drug in terms of selectivity, potency and ADME properties. Collaboration with medicinal chemists is thus needed to improve these properties in iterative cycles of optimization. From a consumer safety perspective, an assessment of possible cardiac toxicity issues related to the use of herbal medicines is warranted. While the carcinogenic potential of herbal drugs has been thoroughly studied, and appropriate measures have been taken, cardiac safety has not yet received sufficient attention. The case of *Evodia* and its main alkaloid DHE shows that investigations into possible hERG liabilities and cardiac safety of other herbal products, in particular alkaloid-containing herbal drugs, are needed.
